# Usability and Concurrent Validity of the Gamified Brain Aging Monitor of Cognition (BAMCOG) for the Self-Monitoring of Perioperative Cognitive Function: A Pilot Study

**DOI:** 10.3390/brainsci15121342

**Published:** 2025-12-18

**Authors:** Mariska E. te Pas, R. Arthur Bouwman, Marcel G. M. Olde Rikkert, Erwin Oosterbos, Pim A. L. Tonino, Steffy W. M. Jansen, Roy P. C. Kessels, Marc P. Buise

**Affiliations:** 1 Department of Anesthesiology, Catharina Hospital, 5623 EJ Eindhoven, The Netherlands; 2 Department of Electrical Engineering, Eindhoven University of Technology, 5612 AP Eindhoven, The Netherlands; 3 Department of Geriatrics, Radboudumc Alzheimer Center, Radboud University Medical Center, 6525 GA Nijmegen, The Netherlands; 4 Department of Cardiology, Catharina Hospital, 5623 EJ Eindhoven, The Netherlands; 5 Department of Biomedical Engineering, Eindhoven University of Technology, 5612 AP Eindhoven, The Netherlands; 6 Department of Geriatrics, Catharina Hospital, 5623 EJ Eindhoven, The Netherlands; 7 Donders Institute for Brain, Cognition and Behaviour, Radboud University, 6525 GA Nijmegen, The Netherlands; 8 Department of Medical Psychology, Radboud University Medical Center, 6525 GA Nijmegen, The Netherlands; 9 Radboudumc Alzheimer Center, Radboud University Medical Center, 6525 GA Nijmegen, The Netherlands; 10 Vincent van Gogh Institute for Psychiatry, 5802 EH Venray, The Netherlands; 11 Department of Anesthesiology and Pain Medicine, Maastricht University Medical Center (MUMC), 6229 HX Maastricht, The Netherlands

**Keywords:** gamification, MoCA, concurrent validity, BAMCOG, mild cognitive impairment

## Abstract

**Objectives**: An increasing number of older adults, including those with mild cognitive impairment, are undergoing cardiac surgery. Despite strong recommendations for preoperative cognitive screening and peri-operative monitoring, routine implementation faces challenges, such as limited time in busy outpatient clinics and lack of patient motivation. To address this issue, gamification and self-administration of cognitive screening using BAMCOG were explored in patients undergoing transcatheter aortic valve replacement (TAVR). **Methods**: A multi-methods prospective repeated-measures within-subject cohort study was conducted between January 2021 and December 2022 to assess usability and concurrent validity. The initial part after game development focused on qualitatively examining the usability of BAMCOG in eight patients, using the System Usability Scale (SUS). The second part, with 40 patients, evaluated concurrent validity by comparing BAMCOG with the widely used Montreal Cognitive Assessment (MoCA). **Results:** The average SUS score was 79.7, indicating good usability. In the preoperative period, the correlation between BAMCOG and MoCA scores was r = 0.33 (*p* < 0.05), which increased to r = 0.59 (*p* < 0.001) on the first postoperative day. **Conclusions**: In conclusion, peri-operative self-monitoring of cognition around a TAVR procedure is feasible, but the concurrent validity of the BAMCOG and the MoCA scores is moderate to low. This warrants further research on gamified cognitive screeners to optimize their use in perioperative cognitive monitoring.

## 1. Introduction

Screening for cognitive impairment does not routinely happen in preoperative evaluation of the surgical patient at the anesthesiology outpatient clinic. This is particularly noteworthy in an aging patient population, because perioperative brain dysfunction is just as significant as other forms of organ system dysfunction for which we routinely conduct preoperative screening and perioperative monitoring [1]. Moreover, in 2012 the American College of Surgeons recommended cognitive assessment in all geriatric surgical patients without known cognitive impairment or dementia to identify risk factors for postoperative delirium, defined as an acute, fluctuating disturbance of attention and awareness after surgery, often with cognitive dysfunction and disorganized thinking [2]. This is important, since a postoperative delirium is independently associated with increased morbidity, cognitive decline up to dementia, and mortality [3]. Recently, Gill et al. published a 1-year mortality rate of 13.4% after major surgery in an older community-dwelling population. In particular, mortality was markedly higher among older persons classified as frail (based on the Fried criteria) or as having probable dementia, defined by the presence of any of the following: (1) a self-report or proxy report of a diagnosis of dementia; (2) a proxy-reported score on the AD8 of ≥2; or (3) a score of ≤1.5 standard deviations below the mean in at least two of three of the following cognitive functioning domains tested with the TICSm and Clock-Drawing Test: orientation, memory, and executive functioning [4,5]. In total, 63% of patients aged 65 or older continue to experience cognitive impairment six months after cardiac surgery, with preoperative cognitive dysfunction being a significant risk factor [6]. In another study, it was noted that only 45% of patients who underwent cardiac surgery, with an average age of 63 years, achieved complete cognitive recovery [7]. Understanding risk factors, such as preoperative cognitive dysfunction, contributes to prevention and directed interventions for the high-risk population [8].

However, detection of Mild Cognitive Impairment (MCI) and other forms of cognitive decline at the preoperative outpatient clinic during a short consultation is challenging, since MCI is defined as objective impairment in cognitive function that does not interfere with independence in daily activities, and may therefore go unnoticed; notably, a recent meta-analysis by Salari et al. reported a prevalence of 23.7% among individuals aged over 60 years [9,10]. The Montreal Cognitive Assessment (MoCA) is a widely used test to screen for MCI and has the best practical features and best psychometric and diagnostic properties to identify patients with MCI [11].

Despite this, lack of time, financial constraints, and limited patient engagement remain key obstacles for routine screening in perioperative care. A self-administered cognitive screener may help to overcome these limitations. However, given that motivation, digital skills, and sustained effort can also be barriers, any self-applied screener must be tested for usability and designed to be user-friendly and engaging. Gamification—the incorporation of game elements into non-game contexts—has been shown to increase motivation and reduce test anxiety, even among older adults, who are increasingly adopting smartphones and tablets regardless of prior experience with touchscreen technology [12,13,14,15]. In this context, remote digital cognitive assessment is expected to play a major role in screening and monitoring cognitive decline in the near future [16]. Against this background, BAMCOG was developed as a gamified, self-administered cognitive screener tailored to the perioperative setting.

In 2013, an online self-monitor for cognitive functioning was developed that makes use of visually attractive, easy-to-instruct puzzle games: the Brain Aging Monitor of COGnition (BAMCOG) [17]. The validation study of the BAMCOG was performed in a cohort of 397 community-dwelling (healthy) individuals with a mean age of 54.9 (SD = 9.6). Yet, there is currently no data or evidence regarding its usability and validity in older patients in the preoperative setting and its suitability as a cognitive screener. Hence, the objective of this study is to assess the usability and concurrent validity of a tablet-based BAMCOG in older and otherwise frail patients undergoing Transcatheter Aortic Valve Replacement (TAVR).

## 2. Materials and Methods

A multi-methods prospective repeated-measures within-subjects cohort study was performed in the Catharina Hospital in Eindhoven, the Netherlands, which is a tertiary referral hospital providing cardiac surgery. Part one involved a small usability study, while part two examined the concurrent validity, as well as the effects of surgery, age, sex, and educational level using a mixed-effects linear model. Two separate cohorts were used to align with the distinct objectives of each part and to facilitate inclusion for part two by omitting the requirement for video recordings.

For both parts of this study, TAVR patients were included, as this patient group consists mostly of older patients with a high risk of preoperative and postoperative cognitive dysfunction [18]. Patients were included if they were (1) 65 years or older, (2) scheduled for a TAVR, (3) able to play games on a tablet after instruction was given, and (4) (near-)native Dutch speaking, because of the Dutch language in the games. Patients were excluded if they (1) had a mental disorder making it impossible to play games on a tablet, (2) had a documented learning disorder (such as dyslexia, dyscalculia, non-verbal learning disorder, or developmental language disorder), (3) consumed ≥15 units of alcohol/week (women) or ≥22 units of alcohol/week (men), or (4) did not sign the informed consent form. An additional inclusion criterion for the usability study was a MoCA score of ≥26 [19,20].

For part one, 11 patients were enrolled between January 2021 and April 2021 and were invited to critically evaluate the usability of the BAMCOG. This small sample size was accepted due to the difficulty in finding patients willing to be filmed during challenging cognitive testing. Therefore, the researcher needed to establish a close relationship with the respondents, enhancing the validity of detailed, in-depth inquiries in a naturalistic setting [21]. Moreover, after examining these 11 patients, no new insights were gained, indicating that the questions had reached a saturation point. Three patients were excluded because of a MoCA score of <26. During a ‘think aloud session’, while using the app, video recordings were made, and patients subsequently rated the BAMCOG according to the System Usability Score (SUS) questionnaire [22]. The SUS is a reliable and validated tool for measuring usability. Participants were asked to answer 10 questions using a five-point Likert scale ranging from ‘strongly agree’ to ‘strongly disagree’, resulting in a score from 0 to 100 (with scores ≥ 68 representing “acceptable” usability levels) [23]. Following the session, patients were prompted to identify three positive and three negative aspects of the BAMCOG. The video recordings were analyzed retrospectively to enhance the BAMCOG for the subsequent phase of the study. If errors were common and the result of a misinterpreted game explanation, these errors were documented. Even explicit suggestions for improvement were documented. These documentations were subsequent arranged in order of appearance. This sequence was followed in making minor adjustments to the games, primarily in the wording of the game instructions, without modifying the content or structure of the games.

For the second part of this study, a new cohort of 53 consecutive patients scheduled for a TAVR were enrolled between July 2021 and December 2022. At least one week before their first preoperative appointment with the cardiologist, patients were identified and contacted by the researcher.

**Figure 1:**
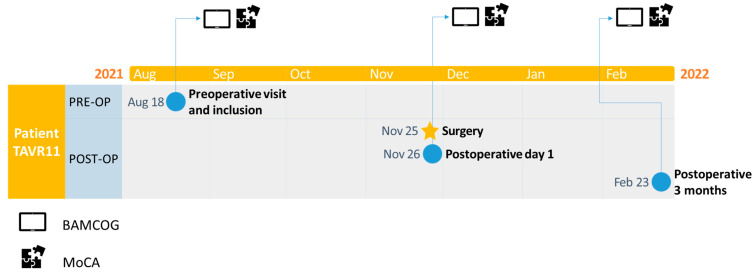
Example timeline of study patient in part two.

**Figure 2:**
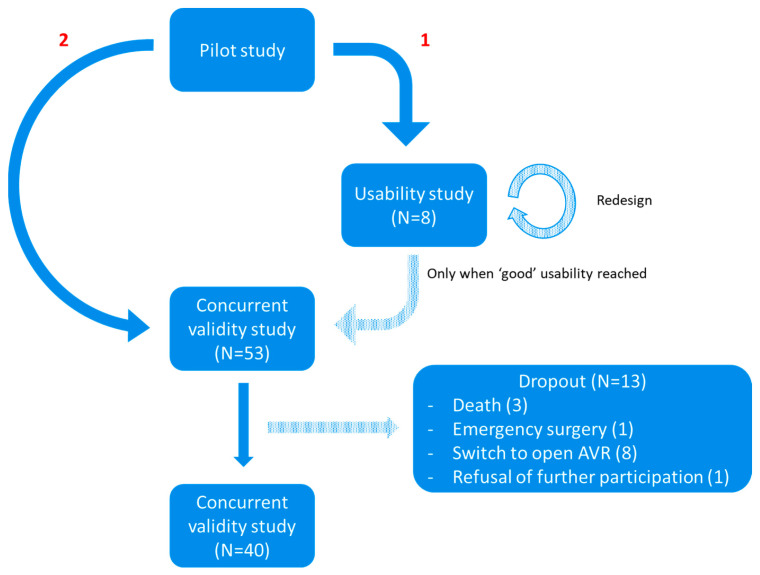
Flowchart study design. ‘1’ = Usability study. ‘2’ = Concurrent validity study.

At the preoperative appointment, patients completed the BAMCOG and the MoCA (Dutch V8.1) as baseline measurements. On the first postoperative day, they underwent a repetition of the BAMCOG and MoCA (Dutch V7.2), followed by another repetition of the BAMCOG and MoCA (Dutch V7.3) three months after TAVR. Alternate forms were used for the repeated cognitive tests. Figure 1 exemplifies the timeline of one study patient. During the study, 13 patients dropped out, due to death after surgery (N = 3), emergency surgery (N = 1), technical inability to place TAVR and a consequent switch to open Aortic Valve Replacement (AVR) (N = 8), and refusal of further participation (N = 1) (Figure 2).

No sample size calculation was made for the first part of this study, as the sample size for this explorative study was based on expert opinion. For the second part, minimum sample size was estimated at 47, based on an α of 0.05 and a power (1 − β) of 0.8, with a hypothesized correlation between BAMCOG and MoCA of 0.4. Taking into account a dropout of 15%, we included 53 patients.

Descriptive data are presented as means and standard deviations (SD), and medians and interquartile ranges (IQR). Correlations between MoCA scores and BAMCOG scores were assessed using Pearson product moment correlation coefficients. Linear mixed-effects analysis was performed to explore the longitudinal nature of our data, using R Studio (version 2024.04.2) and SPSS (version 29).

### 2.1. Montreal Cognitive Assessment [24]

The Montreal Cognitive Assessment (MoCA V8.1, 7.2 & 7.3) is short multi-domain cognitive screen developed for detecting mild cognitive impairment [24]. It can be administered in approximately 10–15 min, results in a score of 0–30 (low-high cognitive functioning), and consists of items assessing memory, visuospatial abilities, executive functions, attention, concentration, working memory, language and orientation in time and place [25]. In an older memory clinic population the MoCA’s sensitivity for detecting MCI ranges from 81 to 93% and the specificity from 74 to 89% [11,26,27,28].

### 2.2. BAMCOG

**Figure 3:**
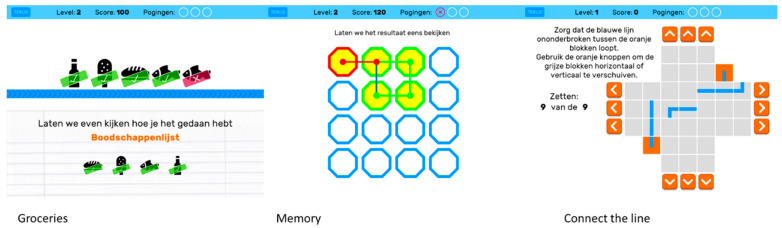
Screenshot BAMCOG Games. Groceries is a game that assesses working memory by asking the participant to keep a short grocery list active in working memory and identify items from this list presented on a conveyer belt under time pressure. The memory game refers to a visuospatial working memory task that assesses sequences of patterns presented in a 5 × 5 grid, which have to be repeated in the same order. Connect-the-line refers to a game that measures visuospatial planning, in which participants have to manipulate individual blocks to establish a uninterrupted path from start to finish. Translation of Dutch wording: “Pogingen”= “Attempts”, “Laten we even kijken hoe je het hebt gedaan” = “Let’s take a look at how you did”, “Boodschappen”= “Groceries”, “Laten we het restultaat eens bekijken” = “Let’s take a look at the result”, “Zorg dat de blauwe lijn ononderbroken tussen de oranje blokken loopt. Gebruik de oranje knoppen om de grijze blokken horizontaal of verticaal te verschuiven” = “Make sure the blue line runs continuously between the orange blocks. Use the orange buttons to move the gray blocks horizontally or vertically”, “Zetten” = “Number of moves”, “9 van de 9” = “9 out of 9”.

The BAMCOG was originally developed as an online self-monitor for cognitive functioning for use on a laptop or personal computer [17]. For this study, the BAMCOG was newly programmed for perioperative use as an application on a tablet (iPad mini, model A1489, 7.9-inch display, 1536 × 2048 pixels, Apple Inc., Cupertino, CA, USA), using Unity development software (version 2019.4 LTS) [29], which can be used to create games for iOS and Android. The new app consists, like the originally BAMCOG, of three games (Figure 3). The first game, called Groceries, assesses working memory and consists of a to-be-remembered grocery list presented on the screen. After 1 s, a conveyer belt, with groceries on it, appears and participants need to select the to-be-remembered products. The maximum score for this game is 590 points. The second game, called Memory, assesses visuospatial short-term memory. Here, visual patterns are presented in a serial way in a 5 × 5 grid. After the visual pattern dissolves, participants are asked to reproduce this pattern in the exact same order as it initially appeared on screen. Maximum score for this game is 620 points. The third game, called Connect the Line, measures planning, as part of executive function. It starts with presenting the participant with a scrambled path. The participants task is to unscramble the path by sliding columns and rows in the correct order so a pawn can move from start to finish unobstructed. Maximum score for this game is 80 points. Every game started with a practice run.

Because of the wide range of scores in the different BAMCOG games, normalized scores were calculated. The overall score is represented as the average of the percentages achieved in the three games. For example, one patient obtained scores of 420, 350, and 40 points in Groceries, Memory, and Connect the Line, respectively. These translate to percentages of 71% (420 out of 590), 56% (350 out of 620), and 50% (40 out of 80). The average percentage of these values is 59%, serving as the total BAMCOG score for this patient.

## 3. Results

**Figure 4:**
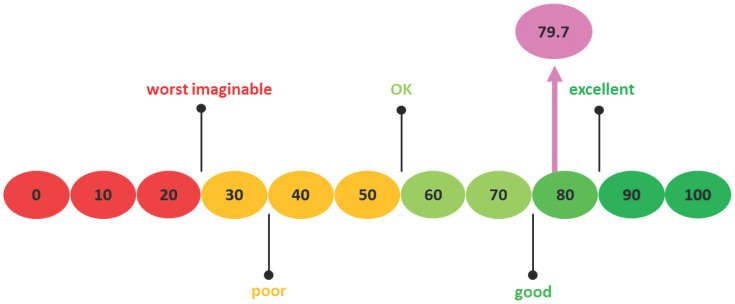
System Usability Scale Scoring with an arrow indicating the SUS score of this study.

The data exhibited skewness and kurtosis values within the range of ±1, indicating that the distribution falls within normality. During the first part of this study, eight patients completed the SUS questionnaire, seven of whom were men. Mean age was 86 (SD 3.9). The mean SUS score was 79.7 (SD 9.0), which can be considered acceptable (Figure 4). The mean MoCA score was 26.3 (SD 0.7) (Table 1). In the second part of the study, 40 patients were included in the correlation analyses, 22 of whom were men. Age ranged from 66 to 90, with a mean of 79.2 years (SD 6.0). The mean level of education was 4.4 (SD 1.8), indicating an educational level ranging from low to average, as per Verhage’s classification of Dutch education levels [30]. Twelve patients received general anesthesia during the TAVR and 28 patients received local anesthesia (Table 1).

**Figure 5:**
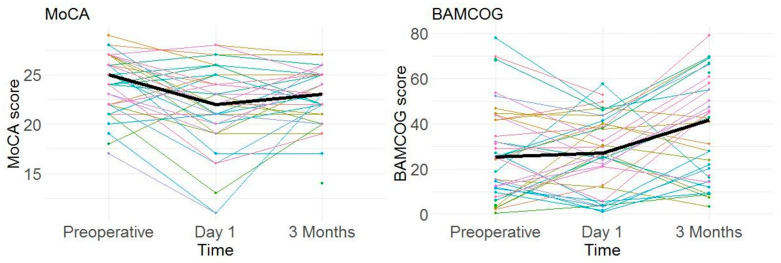
Individual MoCA and BAMCOG scores over time (i.e., preoperative, day 1, and 3 months). Individual MoCA scores are displayed on the left and individual BAMCOG scores on the right, with the mean for each represented by the black line. Individual scores are shown by the different colored lines.

**Figure 6:**
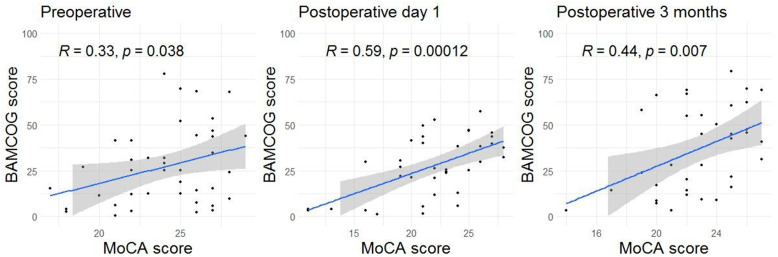
Scatter plots showing the correlations between the MoCA and BAMCOG over time. Each dot represents an individual correlation. The shaded area indicates the 95% confidence interval, and the thick line represents the mean.

Mean preoperative, 1-day and 3-month postoperative MoCA scores were 24.1 (SD 3.1), 21.8 (SD 4.4) and 22.8 (SD 3.0), respectively. Mean preoperative, 1-day and 3-month postoperative normalized BAMCOG scores (% correct) were 27.3 (SD 21.2), 27.4 (SD 16.4) and 37.1 (SD 22.9), respectively (Figure 5). The correlation coefficient of the preoperative, 1-day postoperative and 3-month postoperative measurements were 0.33 (95CI 0.02–0.58), 0.59 (95CI 0.33–0.77) and 0.44 (95CI 0.13–0.67), respectively (Figure 6). Linear mixed-effects analysis revealed that a higher educational level positively influenced both MoCA and BAMCOG scores, while increasing age negatively impacted MoCA and BAMCOG scores (Table 2).

## 4. Discussion

The results of this study suggest a good usability of the BAMCOG in patients undergoing TAVR. Nonetheless, the BAMCOG shows a low to moderate concurrent validity in comparison with the MoCA (the ‘gold standard’) when monitoring perioperative cognition. While average MoCA scores decreased from preoperative to postoperative and increased from 1 day postoperative to 3 months postoperative, BAMCOG scores showed an increase. In the preoperative assessment, we found a weak positive correlation between BAMCOG and MoCA scores. In the assessments conducted on the first day and three months postoperatively, a moderate positive correlation between BAMCOG and MoCA scores was identified.

On the first postoperative day, the correlation between BAMCOG and MoCA was remarkably higher than the correlation found at the baseline preoperative measurements. The post-operative improvement may result from a practice effect due to playing the games multiple times. We used alternate forms for all games to overcome material-specific practice effects and added a practice level to make the patients familiar with the games. However, we observed that some patients had to get used to playing the games for the first time. On consecutive assessments, most patients were more used to playing and played the games more smoothly as a result.

Despite the limited research conducted on the gamification of cognitive screening, our results are consistent with the findings reported by Lumsden et al. in 2016 [12]. They conducted a systematic review indicating positive participant engagement of gamified tasks. In all studies assessing intrinsic motivation, the utilization of game-like tasks was reported to enhance motivation compared to non-gamified versions. Nevertheless, they observed that in replication studies, particularly those comparing multi-domain gamified tasks with non-gamified tasks, the correlations varied. This suggests that designing multi-domain gamified cognitive tasks, such as BAMCOG, is challenging, and it may necessitate multiple well-powered validation studies to ensure that a gamified task accurately measures its intended outcomes. Moreover, the results from a prior study by Goulart et al. [31] align with this suggestion. They explored the correlation between a digital game (MentalPlus^®^) and a battery of standardized neuropsychological tests in 60 patients before and after surgery. Acceptable correlations were only observed with measures of processing speed, suggesting that their game mainly relies on information-processing speed rather than assesses multiple cognitive domains. The correlation between the BAMCOG and the MoCA, which is a multi-domain cognitive screen, provides evidence that the BAMCOG taps multiple cognitive domains, but future studies comparing the BAMCOG to standardized neuropsychological tests are required to substantiate this.

### 4.1. Limitations

A limitation of this study is the small sample size, which consisted of 40 patients instead of the initially calculated 47. This was caused by a higher dropout rate (25%) compared to the anticipated rate (15%). One plausible explanation for this increased dropout rate is the longer waiting times resulting from the COVID-19 pandemic. Another explanation is that withdrawal from the study, though it occurred in only one patient, was due to the cognitive tests being perceived as exhausting and confronting. Additionally, the surgical procedure for eight patients was changed from TAVR to sternotomy due to technical failures that could not have been predicted prior to inclusion. Fortunately, this small sample size does not impact our usability analyses. However, the reduced and relatively homogeneous sample limits the external validity of our findings, as the results may not be generalizable to more diverse patient groups with different types of cognitive impairment, comorbidities, or socioeconomic backgrounds. Therefore, the findings should be considered preliminary and require validation in larger and more heterogeneous cohorts. Despite the primary objective of this study being to conduct usability and correlation analysis, the limited sample size prevents the calculation of reference scores and cut-off scores for BAMCOG. As a result, there is currently no clinical interpretation available for BAMCOG scores. Although the reliability of alternate forms of BAMCOG was investigated in the original validation study [17], showing sufficient to good intraclass correlation coefficients (ICCs) for three out of four games, test–retest reliability and internal consistency were not examined in the present study. These aspects are important for fully establishing BAMCOG’s psychometric properties and should be addressed in future research.

Another limitation in this study is that certain patients were unfamiliar with using a tablet, particularly for gaming purposes. For some patients, it was their first experience with a tablet. We chose not to exclude these individuals, as our aim was to gather real-world data for correlation analyses. Additionally, we observed that the overall normalized BAMCOG scores were notably low, suggesting that the current scoring system may not be appropriate for the studied cohort. Therefore, self-monitoring by younger patients around cardiac surgery may be more feasible and may show less practice effects, and thus a better reliability. Currently, a study is underway to investigate this aspect in a broader age-range of patients undergoing AVR surgery.

The practice effect, which is always present in cognitive tests to some extent, introduces a potential source of bias and is another limitation of the BAMCOG. It raises the possibility that improvements due to practice mask other changes [32]. However, a study by Jutten et al. reveals the intriguing observation that reduced practice effects can function as an indicator of cognitive performance and risk of cognitive decline [33]. This should be confirmed in future research.

### 4.2. Recommendations

To mitigate the potential practice effect arising from multiple game sessions in this study, which may have contributed to the low to moderate concurrent validity between MoCA and BAMCOG, it is necessary to improve the clarity of instructions and implement more effective game difficulty levels. Furthermore, studies with larger and more diverse samples are required to conduct a Reliable Change Index (RCI) analysis [34]. This is necessary to determine whether a change in an individual score over time is statistically significant rather than attributable to random measurement error or practice effects alone. To facilitate clinical application, it is also crucial to gather data from large samples, including healthy volunteers, to establish cut-off scores, gather reference values, and compute normative data. Future research should further expand the sample size to improve generalizability, conduct longitudinal studies with longer follow-up to assess long-term usability and validity, and explore the application of BAMCOG in other surgical populations to evaluate its ability to predict patient outcomes, such as postoperative delirium or prolonged hospital stay.

## 5. Conclusions

The BAMCOG is a test battery to monitor cognitive performance in healthy adults in an online setting. Our study results showed a good usability in older patients scheduled to undergo TAVR and low to moderate correlations between the BAMCOG and MoCA. This suggests that self-monitoring perioperatively in cardiac surgery is possible in most older patients, but that clinically relevant alterations in postoperative cognitive function may not yet be reliably and validly captured with BAMCOG, making its application in clinical practice not yet feasible. Although the potential value of tools using gamification approaches for cognitive screening and self-monitoring are promising, further research on the use of gamified cognitive screeners is still needed.

## Figures and Tables

**Table 1:** Baseline characteristics, first part and second part.

Characteristic	First Part, N = 8 (%)	Second Part, N = 40 (%)
Male	6 (75)	22 (55)
Mean age in years (SD)	86.0 (3.9)	79.2 (6.0)
65–69	-	3 (7.5)
70–74	-	5 (12.5)
75–79	-	12 (30)
80–84	3 (38)	11 (27.5)
85–89	4 (50)	8 (20)
90–94	1 (12)	1 (2.5)
Mean MoCA score (SD)	26.3 (0.7)	-
Mean SUS score (SD)	79.7 (9.0)	-
General Anesthesia	-	12 (30)
Local Anesthesia	-	28 (70)
ASA 3	-	11 (27.5)
ASA 4	-	29 (72.5)
Mean level of education (SD) *	-	4.4 (1.8)
Low (1–4)	-	15 (37.5)
Average (5)	-	14 (35)
High (6–7)	-	11 (27.5)

SD = Standard Deviation, ASA = American Society of Anesthesiologists, SUS = System Usability Scale. * [30].

**Table 2:** Mixed-effects linear model for part two.

	Estimate	95%CI	*p*-Value
MoCA			
Intercept	26.3	23.5–29.1	<0.001
1 day postoperative	−2.5	−2.4–0.3	0.012
3 months postoperative	−1.3	−3.8–−1.3	<0.001
Male	−1.1	−2.8–0.5	0.181
Average level of education *	1.5	−0.3–3.3	0.093
High level of education *	3.7	1.7–5.7	<0.001
Mean age (years)			
70–74	−2.3	−5.8–1.2	0.191
75–79	−2.3	−5.4–0.9	0.153
80–84	−3.7	−6.8–−0.6	<0.05
85–89	−5.3	−8.5–−2.1	<0.05
90–94	−2.7	−8.2–2.8	0.325
BAMCOG			
Intercept	45.6	28.9–62.3	<0.001
1 day postoperative	−0.05	−5.8–5.7	1.0
3 months postoperative	11.2	5.5–16.9	<0.001
Male	8.2	−1.7–18.1	0.103
Average level of education *	0.8	−9.9–11.5	0.881
High level of education *	16.1	4.2–28.0	<0.05
Mean age (years)			
70–74	−31.6	−52.4–−10.8	0.004
75–79	−19.4	−38.0–−0.7	0.043
80–84	−34.6	−52.8–−16.4	<0.001
85–89	−34.2	−53.3–−15.1	<0.001
90–94	−38.5	−71.5–−5.5	0.024

Dependent variables: MoCA and BAMCOG scores. Fixed effects: Time, sex, education level and age. Random effects: Patient ID. Intercept contains preoperative cognitive scores, female gender, low education level, age 65–70 years. * [30].

## Data Availability

The original contributions presented in this study are included in the article. Further inquiries can be directed to the corresponding author.

## Abbreviations

The following abbreviations are used in this manuscript:

AD8	Eight-item Informant Interview to Differentiate Aging and Dementia
BAMCOG	Brain Aging Monitor Cognitive Assessment Battery
CI	Confidence Intervals
MCI	Mild Cognitive Impairment
MoCA	Montreal Cognitive Assessment
SD	Standard Deviation
SUS	System Usability Scale
TAVR	Transcatheter Aortic Valve Replacement
TICS-M	Modified Telephone Interview for Cognitive Status
